# Bevacizumab combined with re-irradiation in recurrent glioblastoma

**DOI:** 10.3389/fonc.2022.961014

**Published:** 2022-08-04

**Authors:** Lei She, Lin Su, Chao Liu

**Affiliations:** ^1^ Department of Clinical Pharmacology, Hunan Key Laboratory of Pharmacogenetics, National Clinical Research Center for Geriatric Disorders, Xiangya Hospital, Central South University, Changsha, China; ^2^ Institute of Clinical Pharmacology, Engineering Research Center for Applied Technology of Pharmacogenomics of Ministry of Education, Central South University, Changsha, China; ^3^ Department of Oncology, Xiangya Hospital, Central South University, Changsha, China

**Keywords:** re-irradiation, recurrent glioblastoma, bevacizumab, gross total resection, temozolomide

## Abstract

**Background:**

Glioblastoma is characterized by rich vasculature and abnormal vascular structure and function. Currently, there is no standard treatment for recurrent glioblastoma (rGBM). Bevacizumab (BEV) has established role of inhibiting neovascularization, alleviating hypoxia in the tumor area and activating the immune microenvironment. BEV may exert synergistic effects with re-irradiation (re-RT) to improve the tumor microenvironment for rGBM.

**Purpose:**

The purpose of this study was to evaluate the safety, tolerability, and efficacy of a combination of BEV and re-RT for rGBM treatment.

**Methods:**

In this retrospective study, a total of 26 rGBM patients with surgical pathologically confirmed glioblastoma and at least one event of recurrence were enrolled. All patients were treated with re-RT in combination with BEV. BEV was administered until progression or serious adverse events.

**Results:**

Median follow-up was 21.9 months for all patients, whereas median progression-free survival (PFS) was 8.0 months (95% confidence interval [CI]: 6.5–9.5 months). In addition, the 6-month and 1-year PFS rates were 65.4% and 28.2%, respectively. The median overall survival (OS), 6-month OS rate, and 1-year OS rate were 13.6 months (95% CI: 10.2–17.0 months), 92.3%, and 67.5%, respectively. The patient showed good tolerance during the treatment with no grade > 3 grade side event and radiation necrosis occurrence rate of 0%. Combined treatment of gross total resection (GTR) before re-RT and concurrent temozolomide during re-RT was an independent prognostic factor that affected both OS and PFS in the whole cohort (OS: 0.067, 95% CI: 0.009–0.521, *p* = 0.010; PFS: 0.238, 95% CI: 0.076–0.744, *p* = 0.038).

**Conclusion:**

In this study, re-RT combined with concurrent and maintenance BEV treatment was safe, tolerable, and effective in rGBM patients. Moreover, GTR before re-RT and selective concurrent temozolomide could further improve patient PFS and OS.

## Introduction

Glioblastoma (GBM) is the most common intracranial malignant tumor accounting for more than 50% of all gliomas (1, 2). It is highly aggressive and has poor prognosis, with 5-year overall survival (OS) rate less than 10% (3, 4). The current standard treatment for GBM includes surgical resection, radiotherapy, and concurrent and adjuvant chemotherapy with temozolomide (TMZ), with median progression-free survival (mPFS) of only 6.9 months (5). In addition, approximately 90% of total recurrences occur in the irradiated field (6). Standard treatment options for recurrent glioblastoma (rGBM) include surgery, re-irradiation (re-RT), chemotherapy, tumor-treating fields (TTFs), targeted therapy, and supportive therapy. However, no category 1 recommendation for rGBM has been provided in the current guidelines.

Re-RT is a treatment option for rGBM (7, 8), which involves several radiotherapy techniques such as intensity-modulated radiotherapy (IMRT), brachytherapy, stereotactic radiosurgery (SRS), hypofractionated stereotactic radiotherapy (HFSRT), and conventional-fractionated radiotherapy (CFRT). A secondary analysis of RTOG 0525 showed that upon GBM progression, median overall survival (mOS) was higher in rGBM patients receiving re-RT than in the supportive treatment group (8.2 months vs. 4.8 months) (9). Furthermore, re-RT combined with systemic chemotherapy achieved significantly higher mOS than the supportive treatment group (12.2 months vs. 4.8 months, *p* < 0.05) (9). In a study including more than 300 patients with rGBM, re-RT increased the 6-month progression-free survival (PFS) rate and 1-year OS rate to 28%–39% and 18%–48%, respectively, even without additional chemotherapy (10). Contrastingly, in another retrospective study including 36 rGBM patients, mOS, 1-year OS rate, mPFS after re-RT, and 6-month PFS rate were 17.2 months, 60.4%, 4.4 months and 41.9%, respectively (11). In general, re-RT is a relatively effective treatment strategy for rGBM (8). However, the occurrence rate of radiation necrosis (RN) in SRS could be higher than 30% (12). Therefore, to improve local control and avoid RN, it is necessary to determine optimal dose and target area for Re-RT by evaluating recurrent tumor volume, initial radiotherapy dose, previous radiation doses delivered to organs at risk, and interval between the initial radiotherapy and re-RT.

The pathological hallmarks of GBM include tumor necrosis, vascular proliferation, abnormal vascular structure, and high expression of angiogenic factors, particularly vascular endothelial growth factor (VEGF) (13, 14). VEGF is an important pro-angiogenesis regulator associated with tumor growth and hypoxic tumor microenvironment (15). Furthermore, preclinical studies suggest that abnormal vascular formation could induce an immunosuppressive microenvironment by modulating the maturation, recruitment, adhesion, and trafficking of immune cells through VEGF signaling pathway (16). Besides, there is a proliferation of some immune negative regulation cells involved in VEGF pathway activation such as myeloid-derived suppressor cells (MDSCs) (17). Bevacizumab (BEV), a humanized monoclonal antibody against VEGF, has been confirmed to improve the PFS of rGBM by the BRAIN study and BELOB trial (18, 19). Consequently, BEV was approved by the U.S. Food and Drug Administration (FDA) in 2009. BEV acts by inhibiting neovascularization, reversing the hypoxia condition in the tumor area, and activating the immune microenvironment (20). Moreover, hypofraction radiation could induce more antigen production, whereas radiotherapy upregulates VEGF (21), suggesting potential synergistic effect of combination therapy of BEV and radiation therapy. In addition, BEV has been shown to reduce RN due to radiation-induced vascular dysfunction (22). In one retrospective study, patients showed good tolerance to re-RT and BEV combination therapy with only four patients (7%) experiencing ≥3 grade toxicity (23).

Some recent studies have evaluated the efficacy of re-RT combined with BEV for the treatment of rGBM. In a retrospective study of 35 patients with recurrent high-grade glioma (rHGG) (59% rGBM) treated with BEV combined with re-RT, mPFS, and mOS were 6.7 and 10.5 months, respectively, with no grade 3 toxicity (24). In another retrospective study of 71 rHGG patients (73.2% rGBM) comprising 57 patients receiving BEV in combination with re-RT (36 Gy/18 fractions), both mPFS (5.6 months vs. 2.5 months, *p* = 0.005) and mOS (8.6 months vs. 5.7 months, *p* = 0.003) improved significantly compared with re-RT monotherapy (23). However, most previous studies included rHGG patients rather than rGBM-only patients. Therefore, a secondary analysis of rGBM subgroup has been seldom explored.

Standard rescue treatment for GBM after relapse and consensus on the efficacy of the combination therapy of re-RT and BEV are lacking. Therefore, our study aimed to evaluate the efficacy, tolerance, and safety of re-RT combined with BEV for rGBM patients and to explore optimal comprehensive treatment for rGBM. The findings may provide clinical reference for future treatment of rGBM.

## Methods

### Patient characteristics

This retrospective study included 26 rGBM patients who had received re-RT between November 2019 and June 2021. The study was approved by the Ethics Committee of Xiangya Hospital of Central South University. Specific inclusion criteria were adopted. First, all patients received standard therapy at initial treatment. Generally, after surgical resection, all patients experienced radiotherapy (median dose: 60 Gy; range: 50–60 Gy) and concurrent and adjuvant TMZ chemotherapy. One patient with non-methylated O6-methylguanine-DNA methyltransferase (MGMT) promoter did not receive TMZ treatment. Second, all patients had no prior BEV usage. Third, all patients had experienced at least one recurrence event and underwent at least one surgery for pathological confirmation of GBM. Fourth, all patients received re-RT combined with concurrent BEV therapy and BEV maintenance therapy until disease progression or unacceptable side effects. Fifth, RN had not occurred since initial radiotherapy. Sixth, all patients had experienced relapse diagnosed using the following criteria: (1) histological pathology and (2) dynamic magnetic resonance imaging (MRI) scan change including magnetic resonance spectroscopy (MRS), perfusion weighted imaging (PWI), and multi-disciplinary team (MDT) discussion. Last, all patients had normal blood routine, liver, and renal tests.

Exclusion criteria were as follows: (1) patients with pregnancy, (2) patients with rGBM declining to participate, (3) complications with other malignant tumors or serious diseases, (4) patients with uncontrolled hypertension not responsive to antihypertensive drug treatment, and (5) patients lacking follow-up data. Additionally, patients with grade I and above myocardial ischemia or myocardial infarction, arrhythmia (including QT interval ≥ 440 ms) and class II cardiac insufficiency were excluded. Patients who experienced arteriovenous thrombosis events within 6 months, such as cerebrovascular accidents (including temporary ischemic attacks), deep venous thrombosis, and pulmonary embolism were also excluded.

### Treatment characteristics and plans

All patients received BEV every 2–3 weeks during re-RT at a median dose of 13 mg/kg (range: 10–15 mg/kg). BEV was administered every 3 weeks after re-RT until detection of progressive disease on MRI scan.

The TMZ regimen administered in our study was based on the results of the RESCUE and DIRECTOR studies indicating that rGBM patients with MGMT promoter methylation or tumor recurrence after TMZ suspension for more than 2 months may benefit from re-prescription of TMZ (25, 26). TMZ capsules were administered at a dose of 75 mg/m2/day during re-RT.

**Figure 1:**
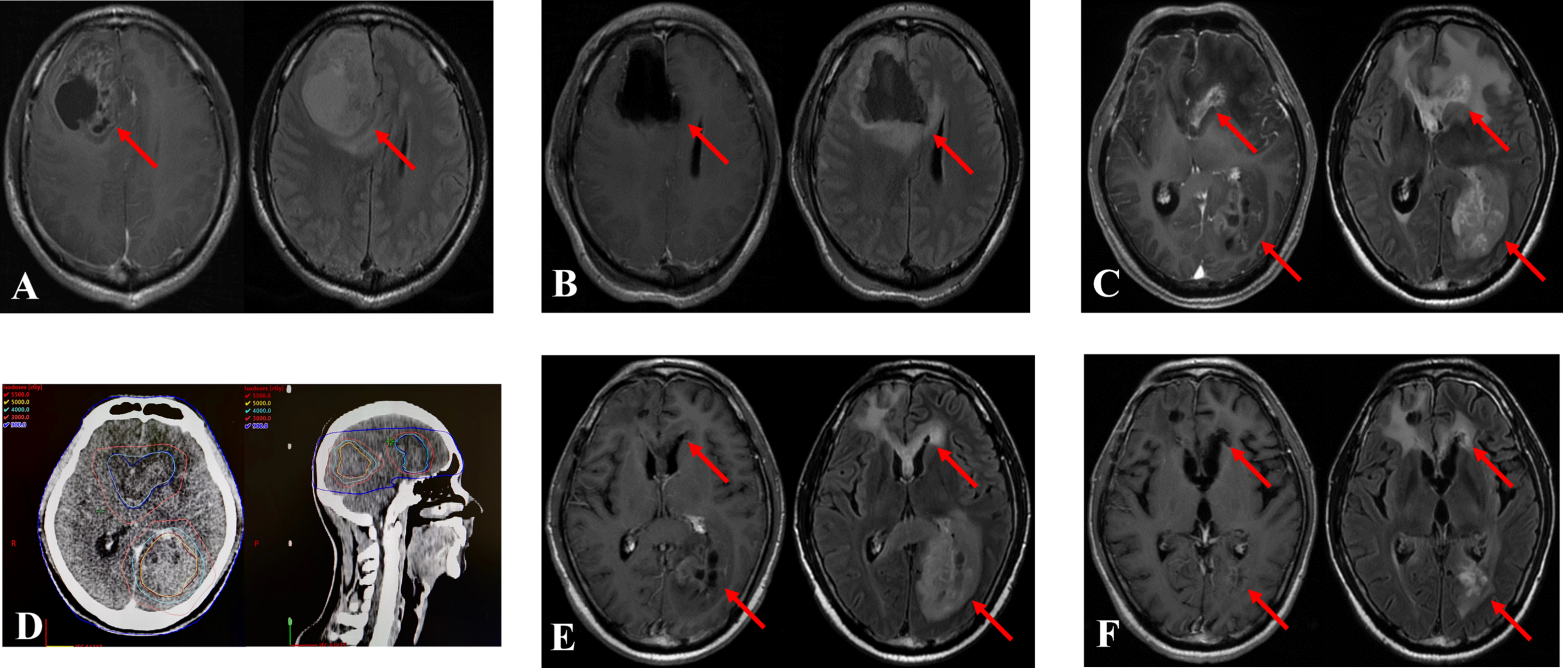
Treatment case of re-irradiation therapy. The two recurrent tumors were diagnosed in a 39-year-old man 13.1 months after the initial operation. The patient underwent re-irradiation of 40 Gy in 20 fractions at *in situ* focus and 50 Gy in 25 fractions at distant locus. **(A)**: Magnetic resonance imaging (MRI) at initial diagnosis. Left: T1 enhanced MRI, Right: T2 fluid enhanced MRI. **(B)**: MRI after the operation. **(C)**: MRI at recurrence. **(D)**: Re-irradiation treatment plan. **(E)**: MRI after re-irradiation. **(F)**: MRI 6 months after re-irradiation. The arrow indicates the tumor area.

During the evaluation of re-RT target area, single dose and total dose, previous radiation doses delivered to organs at risk, Karnofsky performance status (KPS), volume of recurrent tumor, and the interval between the re-RT and initial radiotherapy should be carefully considered. According to European Society for Radiotherapy and Oncology - Advisory Committee on Radiation Oncology Practice (ESTRO-ACROP) (27) guidelines, the target region should be evaluated as follows: clinical target volume (CTV) comprises the recurrent gross tumor volume (GTV) including T1 contrast area and a margin of 0.5–1.0 cm expanded from GTV. Additionally, for secondary glioblastoma, the T2 hyper signaling area should be included as appropriate when evaluating re-RT target area. The radiation doses delivered to organs at risk was determined according to a study by the University of Michigan (28). To avoid radiation damage, the single fraction dose and total dose were set at 3–3.5 Gy and 30–35 Gy, respectively, for a small recurrent tumor volume. However, for a large recurrent tumor volume, the recommended conventional fractionation was used, consisting of fractional dose of 2 Gy and total dose of 40–54 Gy (**Figure 1**).

### Patients assessment

To assess clinical neurological functions, physical examination and head MRI scanning were performed every 2 to 3 months after the completion of re-RT. Efficacy was evaluated following the Response Assessment in neuro-Oncology (RANO) criteria (29). The time of recurrence was determined using dynamic monitoring of MRI changes and other functional MRI examinations such as MRS and PWI. If MRI indicated possibility of tumor relapse or recurrence, MDT discussion was held. Hematological and non-hematological toxicities were classified according to the Common Terminology Criteria for Adverse Events (CTCAEs) version 5.0.

### Statistical analysis

PFS and OS after recurrence were calculated using the Kaplan–Meier method. PFS and OS were defined for the period from the initiation of re-RT to disease progression or death from any cause or the last day of follow-up. The Kaplan–Meier method was used for univariate analysis. Backward stepwise multivariate analysis was performed using Cox’s proportional hazard model. All values with *p* < 0.05 were considered statistically significant. SPSS software (version 25; IBM Corp.) was used for all statistical analyses.

## Results

A total of 26 patients comprising 14 men (53.8%) and 12 women (46.2%) with a median age of 40.5 years old (range: 26–68 years old) were enrolled in this study. All patients received re-RT combined with concomitant and maintenance BEV therapy. **Table 1** summarizes the clinical features of all patients. In total, 13 patients (50%) initially diagnosed with the World Health Organization (WHO) grades II–III glioma developed GBM at recurrence, as confirmed using surgical pathology. Among them, 18 (69.2%), five, and three patients had their first, second, and third recurrence, respectively. Before re-RT, 17 patients (65.4%) underwent surgical resection for the recurrent tumor, including eight gross total resection (GTR) and nine subtotal resection (STR). In total, 21 patients (80.8%) received concurrent chemotherapy with TMZ. The median dose of primary radiotherapy was 60 Gy (range: 50–60 Gy). The median time from the first radiotherapy session to re-RT was 30.2 months (range: 5.6–197.7 months), whereas the median equivalent dose in 2-Gy fractions (EDQ2) of re-RT was 46 Gy (range: 32.5–54 Gy).

**Table 1:** Patient characteristics (*N* = 26).

Characteristic	No. of patients	%
Median Age (year) (range)	40.5 (26–68)	
Sex		
Male	14	53.8%
Female	12	46.2%
KPS		
60	3	11.5%
70	11	42.3%
80	12	46.2%
WHO pathological grade at initial diagnosis		
II-III	13	50%
IV	13	50%
Median dose of the first radiotherapy (Gy) (range)	60(50-60)	
Concurrent TMZ treatment during first radiotherapy		
Yes	25	96.2%
No	1	3.8%
Number of cycles of AC at first diagnosis		
<6	2	7.7%
≥6	24	92.3%
Number of recurrences before re-RT		
1st	18	69.2%
2nd	5	19.2%
3rd	3	11.5%
Mode of recurrence		
In-field only	16	61.5%
Out-field with/without in-field	10	38.5%
Surgery before re-RT		
Gross total resection	8	30.8%
Subtotal resection	9	34.6%
No	9	34.6%
Concurrent TMZ with re-RT		
Yes	21	80.8%
No	5	19.2%
MGMT methylation status		
Methylated	13	50%
Unmethylated	13	50%
IDH mutation status		
Mutated	12	46.2%
Wildtype	14	53.8%
Interval between initial radiotherapy and re-RT (months)		
Median(range)	30.2(5.6-197.7)	
<12	4	15.4%
≥12	22	84.6%
Re-RT modality		
CFRT	22	84.6%
HFSRT	4	15.4%
Median EQD2 of re-RT(Gy) (range)	46(32.5-54)	
Median EQD2_cumulative_ (Gy) (range)	100(92.5-114)	
GTV size (ml)		
<30	10	38.5%
≥30	16	61.5%
Median PTV size (ml) (range)	114.8(11.9-360.1)	

KPS: Karnofsky performance status; WHO: World Health Organization; re-RT: re-irradiation; TMZ: temozolomide; AC: adjuvant chemotherapy; MGMT: O6-methylguanine-DNA methyltransferase; IDH: isocitrate dehydrogenase; CFRT: conventional-fractionated radiotherapy; HFSRT: hypofractionated stereotactic radiotherapy; EQD2: equivalent dose in 2 Gy fractions; GTV: gross tumor volume; PTV: planning target volume.

**Figure 2:**
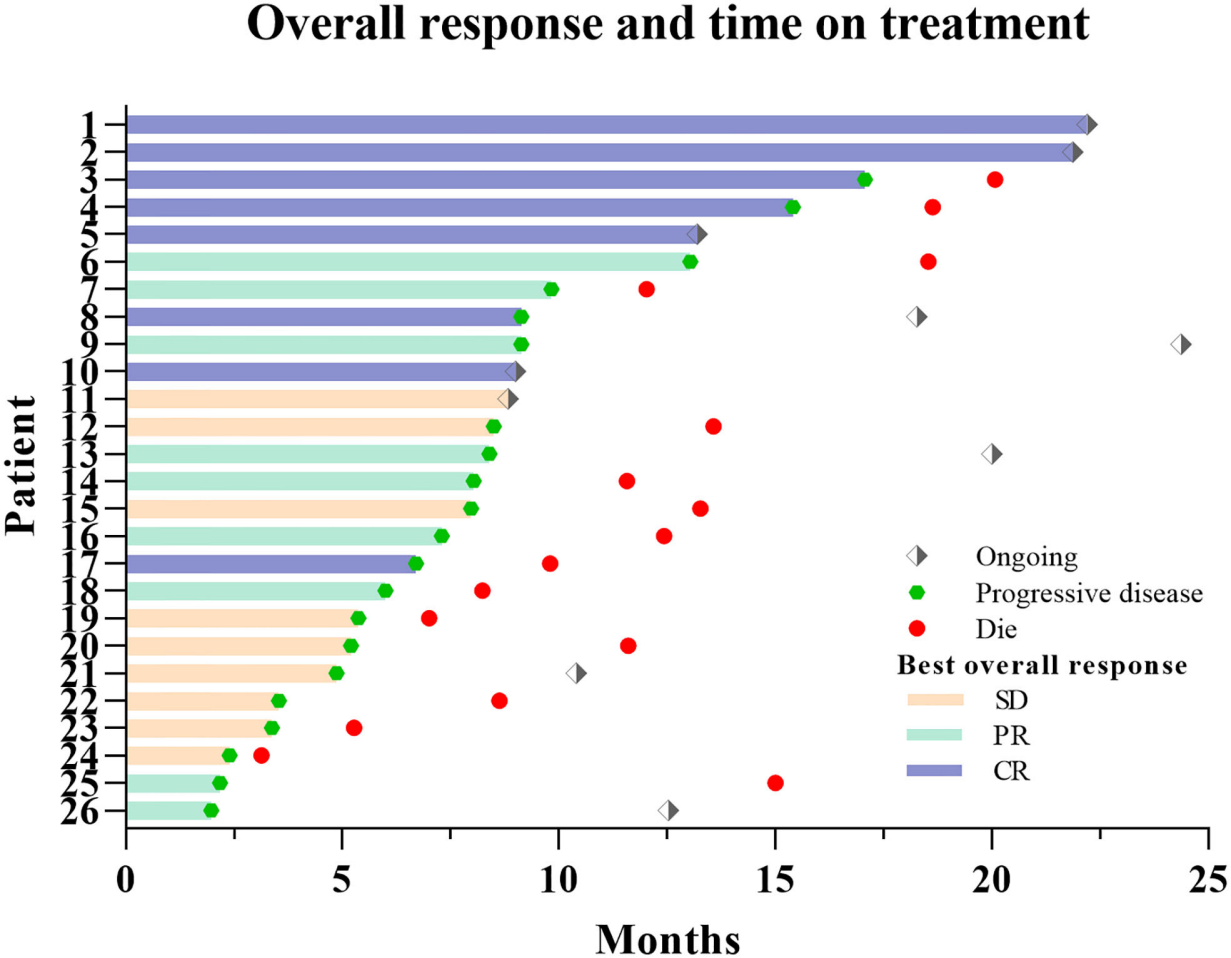
Follow-up of all recurrent glioblastoma (rGBM) patients.

**Figure 3:**
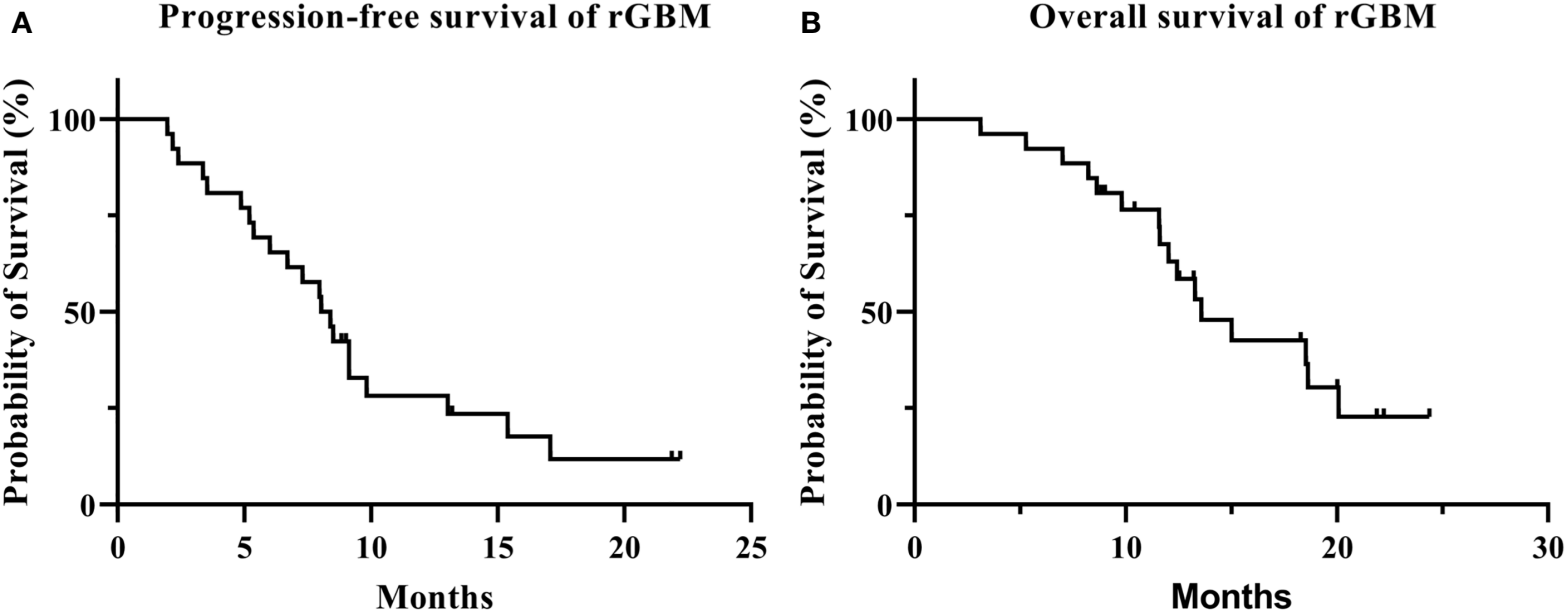
Progression-free survival **(A)** and overall survival **(B)** of recurrent glioblastomas.

The median follow-up was 21.9 months (range: 3.1–24.4 months), with 10 patients (38.5%) alive at last follow-up. Among them, five showed no signs of progress and none was lost to follow-up (**Figure 2**). The median PFS was 8.0 months (95% confidence interval [CI]: 6.5–9.5 months) with 6- and 12-month PFS rate of 65.4% and 28.2%, respectively. The median OS was 13.6 months (95% CI: 10.2–17.0 months) with 6-month and 1-year OS rate of 92.3% and 67.5%, respectively (**Figure 3**).

All patients showed good tolerance during treatment. Two patients (7.7%) had grade 1 symptomatic edema (SE), whereas only one patient (3.8%) exhibited grade 3 hypertension. Additionally, one patient showed grade 3 proteinuria and two (7.7%) showed grade 3 myelosuppression. No patient exhibited toxic side effects beyond grade 4 during the course of the treatment. There was no evidence of RN in any patient during follow-up based on MRI (**Table 2**). KPS increased in 12 patients (46.2%) during the progression-free phase and remained stable among all other prognostic factors (median: 80; range: 70–90). Nine patients (34.6%) received corticosteroids therapy before re-RT, which was reduced or stopped during the combination therapy. No patient required additional corticosteroids therapy during the progression-free phase.

**Table 2:** Safety profile of re-irradiation combined with bevacizumab (*N* = 26), according to CTCAE version 5.0.

Adverse Events	No. of patients (%) and grade
Radiation necrosis	0(0%)
CNS hemorrhage	3(11.5%) grade 1
Epistaxis	4(15.4%) grade 1
Hypertension	7(26.9%) grade 1, 2(7.7%) grade 2, 1(3.8%) grade 3
Deep vein thrombosis	1(3.8%) grade 1
Wound-healing complication	0(0%)
Proteinuria	3(11.5%) grade 1, 1(3.8%) grade 3
Myelosuppression	8(30.8%) grade 1, 2(7.7%) grade 2,2(7.7%) grade 3
Erythra	2(7.7%) grade 1
Symptomatic edema	2(7.7%) grade 1

CTCAE, National Cancer Institute-Common Terminology Criteria Adverse Events; CNS, central nervous system.

**Figure 4:**
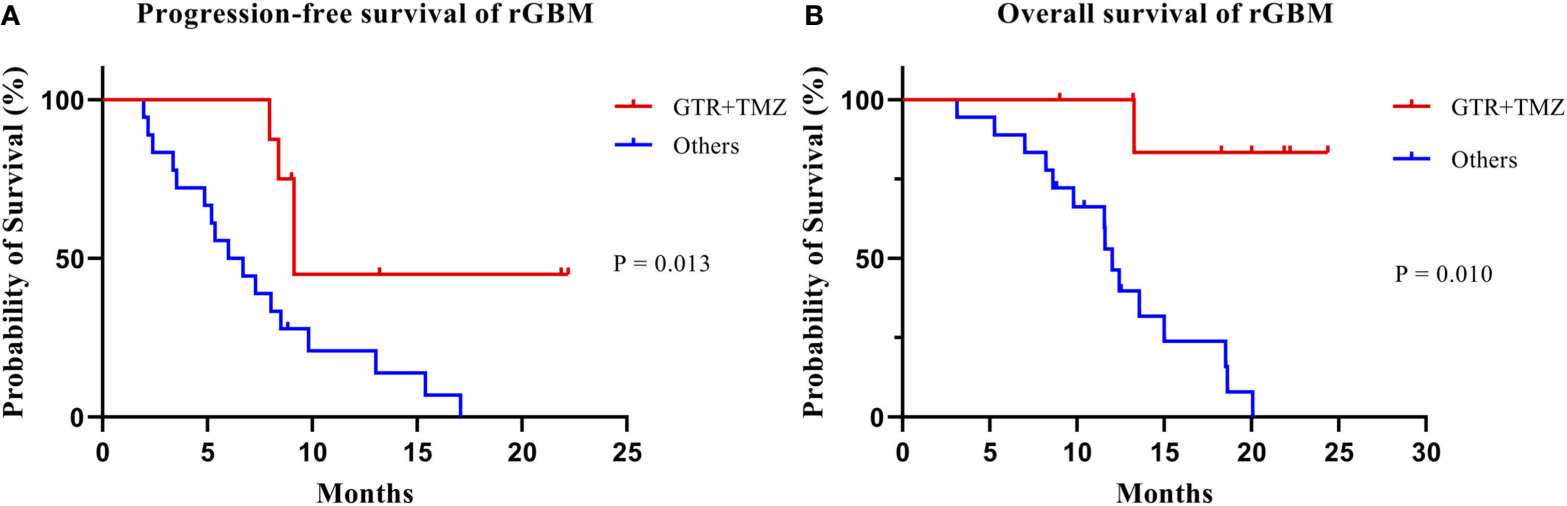
Kaplan–Meier estimates of progression-free survival **(A)** and overall survival **(B)** according to treatment group.

Univariate analysis showed that KPS (*p* = 0.016), GTR before re-RT combined with concurrent TMZ (*p* = 0.016), and the number of recurrent lesions (*p* = 0.014) were significant prognostic factors for OS. On the other hand, sex (*p* = 0.021), the WHO pathological grade at initial diagnosis (*p* = 0.011), GTR before re-RT combined with concurrent TMZ (P = 0.013), concurrent TMZ during re-RT (*p* = 0.004), isocitrate dehydrogenase (IDH) mutation status (*p* = 0.040), pattern of recurrence (*p* = 0.008), GTV (*p* = 0.022), duration between the initial radiotherapy and re-RT (*p* = 0.008), and the number of recurrent lesions (*p* = 0.046) were important prognostic factors affecting PFS (**Table 3**) (**Figure 4**). Significant factors (*p* < 0.05) in univariate analysis results were included in multivariate analysis. In multivariate analysis, GTR before re-RT combined with concurrent TMZ (*p* = 0.010) was independent prognostic factor that significantly affected OS. In comparison, sex (*p* = 0.025), WHO pathological grade at initial diagnosis (*p* = 0.034), and GTR before re-RT combined with concurrent TMZ (*p* = 0.014) were significant independent prognostic factors for PFS (**Table 4**).

**Table 3:** Univariate analysis for OS and PFS.

Variable		1-year OS	*P*-value (Log-Rank)	1year PFS	*P*-value (Log-Rank)
Age	<50 years	60.9%	0.264	22.5%	0.730
	≥50 years	80.0%		37.5%	
Sex	Male	55.6%	0.121	7.1%	**0.021**
	Female	83.3%		55.6%	
KPS	≤70	57.1%	**0.016**	21.4%	0.078
	>70	80.2%		35.0%	
WHO pathological grade at initial diagnosis	II-III	65.8%	0.964	51.3%	**0.011**
	IV	68.4%		7.7%	
GTR before re-RT combined with concurrent TMZ	Yes	83.3%	**0.001**	45.0%	**0.013**
	No	53.0%		20.8%	
Concurrent TMZ during re-RT	Yes	70.2%	0.188	34.9%	**0.004**
	No	53.3%		0%	
MGMT methylation status	Meth	75.2%	0.357	32.3%	0.413
	Unmeth	60.6%		23.1%	
IDH mutation status	Mutated	62.5%	0.818	46.7%	**0.040**
	Wild-type	70.7%		14.3%	
Re-RT dose (EQD2)	<50 Gy	56.5%	0.077	21.2%	0.089
	≥50 Gy	87.5%		41.7%	
Mode of recurrence	In-field only	72.1%	0.060	36.5%	**0.008**
	Others	60.0%		10%	
GTV	<30ml	70.0%	0.561	10%	**0.022**
	≥30ml	72.9%		40.2%	
Interval between initial radiotherapy and re-RT	<12 months	100%	0.817	0%	**0.008**
	≥12 months	61.8%		33.3%	
The number of recurrent lesions	single	76.1%	**0.014**	32.9%	**0.046**
	multiple	42.9%		14.3%	
Number of recurrences before re-RT	1	77.8%	0.194	38.1%	0.390
	More than 1	43.8%		0%	

OS, overall survival; PFS, progression-free survival; KPS, Karnofsky performance status; WHO, World Health Organization; GTR, gross total resection; TMZ, temozolomide; Re-RT, re-irradiation; MGMT, O6-methylguanine-DNA methyltransferase; IDH, isocitrate dehydrogenase; EQD2, equivalent dose in 2 Gy fractions; GTV, gross tumor volume. Significant factors in bold.

**Table 4:** Multivariate analysis for OS and PFS.

Variable	OS		PFS	
	*P*-value	Hazard ratio (95% CI)	*P*-value	Hazard ratio (95% CI)
Sex (Female vs. Male)	**/**	**/**	**0.019**	**0.273(0.092-0.807)**
KPS	Exclude prior to last step	**/**	/	**/**
WHO pathological grade at initial diagnosis (II-III vs. IV)	/	/	**0.034**	**0.353(0.135-0.922)**
GTR before re-RT combined with concurrent TMZ (Yes vs. NO)	**0.010**	**0.067(0.009-0.521)**	**0.014**	**0.238(0.076-0.744)**
Concurrent TMZ during re-RT (Yes vs. NO)	/	/	Exclude prior to last step	/
IDH mutation status	/	**/**	Exclude prior to last step	/
Mode of recurrence	/	/	Exclude prior to last step	**/**
GTV	/	/	Exclude prior to last step	**/**
Interval between initial radiotherapy and re-RT	/	/	Exclude prior to last step	**/**
The number of recurrent lesions	Exclude prior to last step	**/**	Exclude prior to last step	/

OS, overall survival; PFS, progression-free survival; 95% CI, 95% confidence interval; KPS, Karnofsky performance status; WHO, World Health Organization; GTR, gross total resection; TMZ, temozolomide; Re-RT, re-irradiation; EQD2, equivalent dose in 2 Gy fractions; IDH, isocitrate dehydrogenase; GTV, gross tumor volume. Significant factors in bold.

## Discussion

GBM is a common primary malignant tumor of the central nervous system with a strong invasion capability and poor survival outcomes (30). Most patients relapse within 1 year after surgery (5, 31). Treatment options for rGBM include surgery, re-RT, systemic therapy, TTF, targeted therapy, and supportive therapy. However, there is no standard treatment for rGBM.

Re-RT is feasible for patients with rGBM in various age groups (32). A review of re-RT studies on rGBM published from 2005 to 2020 reported that mOS and mPFS of 7.5–13 months and 4.4–6 months, respectively, for patients treated with SRS compared with 7.3–12.5 months and 2.6–7.5 months for those treated with HFSRT (8). In addition, mOS and mPFS of patients treated with CFRT were 6.7–11.5 months and 2.5–5 months, respectively, and patients treated with CFRT were at the lowest risk of developing RN (0%–6.9%) (8). In a systematic review and meta-analysis of 50 studies including 2,095 patients with rGBM (patients included in 38 studies received re-RT alone) by Kazmi et al., the 6-month PFS rate of rGBM after re-RT, 12-month PFS rate, 6-month OS rate, 12-month OS rate, and incidence of grade 3 toxicity were 43%, 17%, 73%, 36%, and 7%, respectively (33). Some retrospective re-RT studies involving patients with rHGG suggested no significant difference in mOS between patients treated with CFRT, HFRT, or SRS (34, 35). However, to avoid RN in clinical practice, optimal choice of radiation techniques and dose is achieved based on the volume of the recurrent tumor, the interval between the initial radiotherapy and re-RT and the dose in organs at risk.

Generally, VEGF is overexpressed in patients with GBM and is associated with poor prognosis (36). VEGF is upregulated during radiotherapy (21), and antiangiogenic drugs could reduce the formation of new blood vessels, thereby inhibiting tumor growth (37). In addition, BEV normalizes blood vessels and alleviate hypoxia in the tumor area, increasing its sensitivity to radiation therapy (38–40). Furthermore, it reduces the permeability of capillaries, decreasing the RN risk (41). BEV could reverse immunosuppression microenvironment and activate the immune process, thereby killing tumor cell. In sum, given the synergistic antitumor mechanisms and anti-RN efficacy, treating rGBM patients with BEV combined with re-RT seems clinically supported.

At present, most studies on re-RT+BEV therapy for recurrent glioma are retrospective and mostly include rHGG subjects, including patients with WHO grades III and IV rHGG. Few studies have included rGBM-only patients. In a systematic review of 34 studies (including a total of 1,399 rHGG patients comprising 79.8% rGBM patients), BEV treatment was the only prognostic factor for improving OS in a multivariate analysis of 445 patients (76% of the patients with rGBM) treated with re-RT+BEV (42). Additionally, OS was improved by 2.5 months (*p* = 0.041). However, PFS did not show any significant improvement (*p* = 0.099), whereas patients treated with BEV had significantly lower incidence of RN (2.2% vs. 6.5%, *p* < 0.001) (42). In addition, no significant differences in OS and PFS were found between the three radiotherapy modalities (SRS, HFSRT, and CFRT) (42). Furthermore, multivariate analysis showed that the pathological type was not a significant prognostic factor for survival. However, in this study, all analyses were based on the whole cohort and no further stratification of rGBM was explored.

Other studies evaluating rGBM showed that patients receiving re-RT+BEV treatment had mPFS of 5.1–7.3 months and mOS of 9.3–13.3 months (**Table 5**) (23, 43–47). The results of RTOG1205 study found no significant difference in mOS between “only BEV” group and patients receiving HFRT (35 Gy/10 F) combined with BEV treatment (10.1 months vs. 9.7 months, 0.98, 95% CI: 0.7–1.38, *p* = 0.5) (43). However, the 6-month PFS rate improved substantially (54% vs. 29%, 0.42, 95% CI: 0.34–0.5, *p* = 0.001), patients showed good tolerance, and the rate of occurrence of acute adverse events above grade 3 was 5% (43). Similarly, Flieger et al. showed that among the 45 rGBM patients who received re-RT, mPFS of those who received concurrent BEV therapy was significantly longer than those who did not (5.1 months vs. 3.4 months, *p* = 0.06), but the mOS was not significantly different between them (9.3 months vs. 6.1 months, *p* = 0.27) (23). However, Cuneo et al. showed that re-RT combined with BEV significantly prolonged both mPFS and mOS (mPFS: 5.2 months vs. 2.1 months, *p* = 0.014; mOS: 11.2 months vs. 3.9 months, *p* = 0.005) (46). Surprisingly, in our study, re-RT combined with concurrent and maintenance BEV, with a median follow-up of 21.9 months had mPFS of 8.0 months, 6-month PFS of 65.4%, 1-year PFS of 28.2%, mOS of 13.6 months, OS rate of 92.3%, and 1-year OS of 67.5%. Unfortunately, we did not include a control group of patients who had received only re-RT or BEV alone. Our results, compared with those reported by Kazmi et al. (33), suggested that combining BEV with re-RT may yield better results than re-RT alone. Additionally, our study suggested a larger survival benefit to patients compared to the studies reviewed here that administered re-RT+BEV. In comparison with these studies, in our study, BEV was used in both concurrent and maintenance modalities. Schnell et al. found that among rHGG patients who had received re-RT, patients who had received concurrent and maintenance BEV treatment exhibited significantly longer mOS than concurrent BEV only group (13.1 months vs. 8.0 months, *p* = 0.006). Multivariate analysis showed that concurrent and maintenance BEV treatment were independent prognostic factors significantly affecting OS (*p* = 0.045) (48). Therefore, concurrent and maintenance BEV therapy may be important factors affecting survival.

**Table 5:** Re-irradiation combined with bevacizumab for recurrent glioblastomas: literature review.

Reference	Pts	Modality	Median dose/fractions	Median EQD2 (Gy)	Median Cumulative EQD2 (Gy)	Systemic therapy	Median PFS (months)	Median OS (months)	RN (%)
Tsien et al., 2019 (43)	170	HFSRT	35 Gy/10 f	48.1	108.1	Bev + HFSRT vs. Bev	54% vs. 29% at 6 m *P* = 0.001	10.1 vs. 9.7 *P* = 0.5	0
Gutin et al., 2009 (44)	20	HFSRT	30 Gy/5 f	60	120	Concurrent and maintenance Bev	7.3(4.4-8.9)	12.5(6.9-22.8) 54% at 1 year	0
Youland et al., 2017 (45)	26	HFSRT	35-40 Gy/10 f	48.2	108.2	Concurrent Bev	6.7	10.6	0
Cuneo et al., 2012 (46)	49	SRS	15 Gy	63.8	123.8	Concurrent or maintenance Bev vs. Other therapy	5.2 vs. 2.1 *P* = 0.014	11.2 vs. 3.9 50% vs. 22% at 1 year *P* = 0.005	5% vs. 19%
Morris et al., 2019 (47)	45	SRS	17 (13–24) Gy	80.7	140.7	Maintenance Bev	5.2	13.3	0
Flieger et al., 2014 (23)	45	CFRT	36 Gy/18 f	36	96	Concurrent Bev vs. Other therapy	5.1 vs. 3.4 *P* = 0.06	9.3 vs. 6.1 *P* = 0.27	0

OS, overall survival; PFS, progression-free survival; EQD2, equivalent dose in 2 Gy fractions; RN, radiation necrosis; Bev, Bevacizumab; HFSRT, hypofractionated stereotactic radiotherapy; SRS, stereotactic radiosurgery; CFRT, conventional-fractionated radiotherapy.

In addition to potentially improving PFS and OS, BEV in combination with re-RT could also reduce RN and dependence on corticosteroids, thereby improving the patients’ quality of life. In Fleischmann et al.’s study, of the 161 rHGG patients, 124 with rGBM were treated with concurrent BEV with re-RT and 37 received re-RT only (49). Multivariate analysis results showed a decrease in the incidence of RN/SE and 1-year incidence (21.8% vs. 37.8%, *p* = 0.025 and 23.9% vs. 54.1%, *p* = 0.013, respectively) in “no BEV concomitant to reRT” group and lack of BEV was the only risk factor for RN/SE (*p* = 0.026) (49). Here, we confirmed that BEV was significantly associated with a reduced incidence of RN and SE. Levin et al. conducted a randomized controlled clinical trial for BEV in 14 patients with typical RN in the central nervous system confirmed by imaging or biopsy evidence and with progressive neurological symptoms or signs (22). All patients treated with BEV showed improvement in neurological symptoms, thereby providing level I evidence for BEV efficacy in treating RN in the central nervous system (22). Similarly, other studies reported that BEV reversed RN (50, 51). **Table 5** shows that the incidence of RN after re-RT+BEV is 0%–5% in rGBM patients. In our study, after a median follow-up of 21.9 months, the incidence of RN was 0, with two patients (7.7%) developing grade 1 SE, consistent with previous findings. In addition, some studies have shown a statistically significant correlation between RN rate and radiotherapy technology. Analysis of 70 articles (a total of 3,302 rHGG patients) found mean RN rate of 7.1% (95% CI: 6.6–7.7) for FSRT, 6.1% (95% CI: 5.6–6.6) for SRS, and 1.1% (95% CI: 0.5–1.7) for CFRT (52). In addition, each 1 Gy increase in total equivalent dose in 2 Gy fractions (EQD2) increased the RN rate by 0.1% after re-RT (52). Some studies suggest that to reduce RN, the cumulative EQD2 should be less than 100 Gy (53–55). In our study, most patients (84.6%) were treated with CFRT, yielding a median cumulative EQD2 of 100 Gy and no patient with RN, which may be attributed to the combination of re-RT and BEV, optimal technology use and appropriate dose.

Corticosteroids are often used to reduce peritumoral angiogenic edema and relieve symptoms associated with high intracranial pressure (56). Long-term use of corticosteroids is associated with many side effects. Therefore, it is recommended to use the lowest dose of corticosteroids for the shortest period. BEV can reduce vascular leakage and intracranial pressure due to brain tumors and decrease vasogenic edema, reducing corticosteroid administration (57). Studies showed that BEV could reduce the use of corticosteroids in rGBM patients, improving the quality of life of patients (58, 59). In our study, patients gradually reduced corticosteroids dose and discontinued during the progression-free periods with no patients requiring additional corticosteroids therapy. KPS increased in 46.2% of patients but remained stable in others, which was consistent with previous findings.

Although rGBM patients are presumed to tolerate the combination treatment, it is not clear which subgroup maybe benefit most from combination therapy. In this study, we sought to identify the significant prognostic factors affecting survival when using combination therapy.

Besides different treatment regimens for rGBM, no prognostic factor (including age, sex, KPS, recurrent tumor volume, IDH1 mutation status, and MGMT promotor methylation status) has been reported to exert a significant impact on OS and PFS (60). A review of the literature on re-RT+BEV treatment for rHGG showed that age, sex, KPS, the WHO histological grade, resection extent, the interval between the initial radiotherapy and re-RT, number of recurrence, and pattern of recurrence did not affect PFS and OS (61). However, univariate analysis by Schernberg et al. showed that age <55 years (*p* = 0.024) and EQD2 >50Gy (*p* = 0.046) were significantly correlated with OS, whereas the WHO histological grade, the interval between the initial radiotherapy and re-RT were not significant factors affecting OS (24). However, age ≥55 years (*p* = 0.001) was a risk factor for PFS. In our study, age ≥50 years was not a significant risk factor for OS and PFS, but women showed better PFS than men, which may be due to our small sample size. However, further investigations are needed to verify this relationship. Similarly, in the study by Schernberg et al., WHO histological grade at initial diagnosis was not significantly associated with OS and PFS after re-RT (*p* = 0.104 and *p* = 0.115, respectively), but the WHO histological grading at the time of recurrence rather than preliminary diagnosis was deemed to have predictive effects (24). Our multivariate analysis showed that the WHO histological grade at initial diagnosis could significantly affect PFS (*p* = 0.011). The possible explanation for the discrepancy with the results of Schernberg et al.’s study is that some patients in other studies retained WHO grade III gliomas after progression, and stratified subgroup analysis of grades III and IV was not performed. In our study, 13 patients previously diagnosed with WHO grades II–III gliomas eventually progressed to GBM and had a better survival outcome compared with primary GBM. Some studies suggested that because IDH mutations are more common in secondary glioblastoma, patient survival and prognosis are better than in primary glioblastoma (62, 63), consistent with the conclusions of our study. We also found that the interval between the initial radiotherapy and re-RT, GTV, the number of recurrent lesions, and the pattern of recurrence were all significantly correlated with PFS and KPS, and the number of recurrent lesions was significantly correlated with OS based on univariate analysis results.

In a retrospective study by Kim et al., including 36 rGBM patients who underwent SRS or HFSRT, univariate analysis found that surgical resection was significantly associated with OS (*p* = 0.010) with the extent of resection showing a positive association with OS (*p* = 0.071) (11). A previous study also showed that GTR before radiotherapy (*p* = 0.047) effectively extended the survival in rGBM patients receiving re-RT (64). Since surgical resection can reduce tumor load, it should be considered for rGBM patients before re-RT (preferably GTR) (11, 65). In our study, all patients who received GTR before re-RT also received concurrent chemotherapy with TMZ. Univariate and multivariate analyses revealed that GTR+TMZ was an independent prognostic factor for OS and PFS. However, we could not separately determine the survival benefits of GTR or concurrent TMZ due to the small sample.

The therapeutic value of TMZ in rGBM is not yet known. The RESCUE study showed that in the rGBM rechallenge in TMZ group yielded a 6-month PFS rate of 35.7% and 1-year OS rate of 28.6%, which was higher than that in the early and the extended groups (25). In addition, the DIRECTOR study suggested that after the completion of the standard STUPP therapy, rGBM patients with MGMT promoter methylation could still benefit from repeated TMZ chemotherapy (26). Barney et al. concluded that TMZ combined with re-RT is safe and effective, because it had a median survival of 5.1 to 10.1 months for rGBM patients after combination therapy (66). Patients with concurrent TMZ in our study were characterized by MGMT methylation or no significant TMZ resistance (progression occurred 2 months after TMZ treatment was discontinued). In our univariate analysis, concurrent TMZ was a prognostic factor for PFS (*p* = 0.004). Therefore, selective concurrent TMZ therapy may benefit survival.

There are some limitations to this study. First, due to its retrospective design, our study was prone to selection bias. Second, the number of patients was small, thus a larger sample may be warranted to verify our results. The study also lacked a control group. Furthermore, some heterogeneity existed in treatment options.

## Conclusions

In conclusion, re-RT combined with concurrent and maintenance BEV therapy is safe and well tolerated by patients with rGBM, as evidenced by the significant reduction in the incidence of RN and patient dependence on corticosteroids and improvement in the KPS score of patients during disease progression-free periods. Besides, combination therapy is an effective strategy for rGBM. In our cohort, we obtained mPFS and mOS of 8 months and 13.6 months, respectively. In comparison with other studies, our findings indicate that the improved therapeutic effects may be in part due to GTR before re-RT, selective concurrent TMZ, and maintenance treatment with BEV. Therefore, based on the premise that rGBM patients can tolerate re-RT+BEV therapy, we speculate that GTR before re-RT and concurrent TMZ may further improve the OS and life quality in rGBM patients. However, prospective randomized controlled studies using larger sample sizes are needed to validate the effectiveness of this combination therapeutic strategy. It would also be essential to explore the mechanism underlying the efficacy of the combination therapy in future.

## Data availability statement

The data presented in this study are available on request from the corresponding author. The data are not publicly available due to privacy.

## Ethics statement

The study was conducted in accordance with the Declaration of Helsinki, and approved by the Ethics Committee of Xiangya Hospital of Central South University (No. 202203079). Written informed consent has been exempted.

## Author contributions

LSh: Data curation; Formal analysis; Investigation; Methodology; Writing – original draft; Supervision. LSu: Investigation. CL: Conceptualization; Funding acquisition; Supervision; Writing – review and editing. All authors contributed to the article and approved the submitted version.

## Funding

This study was supported by the Natural Science Youth Foundation of Hunan Province (grant no. 2018JJ3856).

## Conflict of interest

The authors declare that the research was conducted in the absence of any commercial or financial relationships that could be construed as a potential conflict of interest.

## Publisher’s note

All claims expressed in this article are solely those of the authors and do not necessarily represent those of their affiliated organizations, or those of the publisher, the editors and the reviewers. Any product that may be evaluated in this article, or claim that may be made by its manufacturer, is not guaranteed or endorsed by the publisher.
